# The Polymerization Effect on Synthesis and Visible-Light Photocatalytic Properties of Low-Temperature *β*-BiNbO_4_ Using Nb-Citrate Precursor

**DOI:** 10.1186/s11671-015-1165-z

**Published:** 2015-12-01

**Authors:** Haifa Zhai, Jizhou Kong, Anzhen Wang, Hongjing Li, Tiantian Zhang, Aidong Li, Di Wu

**Affiliations:** Henan Key Laboratory of Photovoltaic Materials, College of Physics and Electronic Engineering, Henan Normal University, Xinxiang, 453007 People’s Republic of China; National Laboratory of Solid State Microstructures, Department of Materials Science and Engineering, College of Engineering and Applied Sciences, Nanjing University, Nanjing, 210093 People’s Republic of China; State Key Laboratory of Mechanics and Control of Mechanical Structures, Nanjing University of Aeronautics and Astronautics, Nanjing, 210016 People’s Republic of China

**Keywords:** *β*-BiNbO_4_, Polymerization, Photocatalytic performance

## Abstract

Low-temperature *β*-BiNbO_4_ powders (denoted as Low-*β*) were prepared by citrate and Pechini methods using homemade water-soluble niobium precursors. The addition of ethylene glycol and the resultant polymerization effect on the synthesis and visible-light photocatalytic performance of *β*-BiNbO_4_ powders were fully investigated. The polymerization effect is beneficial to lower the phase formation temperature and obtain smaller particle catalysts. Both methods can synthesize catalysts with excellent performance of visible-light degradation of methyl violet (MV). The Low-*β* BiNbO_4_ powder prepared by citrate method shows better degradation rate of about 1 h to decompose 80 % of MV and also displays good photocatalytic stability. The photodegradation of MV under the visible-light irradiation followed the pseudo-first-order kinetics according to the Langmuir-Hinshelwood model, and the obtained first-order rate constant and half-time are 2.85 × 10^−2^ min^−1^ and 24.3 min, respectively. The better photocatalytic performance of BiNbO_4_ powders prepared by citrate method can be attributed to its smaller band gap and better crystallinity.

## Background

In recent years, environmental pollution, especially organic pollutants, has attracted much attention due to its deleterious effect on human health [1]. TiO_2_, one of the most popular photocatalysts, can solely absorb ultraviolet (UV) light. In order to make good use of solar light source, many visible-light active photocatalysts have been deeply investigated, such as quantum dot-based photocatalysts [2–5]. Among these photocatalysts, much attention has been paid on bismuth-based photocatalytic materials, such as BiOBr [6, 7], Bi_2_O_2_CO_3_ [8], BiVO_4_ [9], BiNbO_4_ [10–12], and BiTaO_4_ [13, 14], due to their excellent photooxidation ability for organic contaminant degradation via a visible-light photocatalytic process.

BiNbO_4_ has orthorhombic *α* and triclinic *β* phases. In general, the low-temperature *α* phase synthesized at 900 °C irreversibly transforms to the high-temperature *β* phase (denoted as High-*β*) at 1020 °C [15]. While in our former work, we first synthesized the low-temperature *β* phase (denoted as Low-*β*) at 700 °C and observed the phase transition from *β* to *α* phase [16]. The visible-light photocatalytic performance test also shows that the Low-*β* exhibits the best photocatalytic efficiency compared with *α* phase and High-*β* [12]. The formation of a pure triclinic phase of BiNbO_4_ at a low temperature of 700 °C can be attributed to the advantage of the citrate method.

The citrate method is a simple way to obtain stable precursors and reactive and stoichiometric fine powders which has been widely used in the fabrication of various simple and complicated oxides [17]. Compared to the citrate method, the Pechini method is superior to obtain a homogeneous multicomponent gel without any phase segregation throughout the processing, which is beneficial to prepared stoichiometric oxides with a much lower temperature and smaller particle size. The difference between these two methods is the introduction of polyhydroxy alcohol, such as ethylene glycol, and the polymerization process between metal citrate and polyhydroxy alcohol. The polymerization results in immobilization of metal complexes in rigid organic polymer nets, thus ensuring the compositional homogeneity [18].

In this work, Low-*β* BiNbO_4_ powders were prepared by citrate and Pechini methods using homemade water-soluble niobium precursors. The addition of ethylene glycol and the resultant polymerization effect on the synthesis and visible-light photocatalytic performance of Low-*β* BiNbO_4_ powders were investigated, including the phase formation process, particle size, optical properties, and the photocatalytic degradation of methyl violet (MV) under visible light.

## Methods

The precursor materials were bismuth nitrate (Bi(NO_3_)_3_·5H_2_O), ammonia, ethylene glycol (EG), and a Nb-citrate (Nb-CA) aqueous solution. The synthesis of water-soluble Nb-CA has been described in detail in a previous work [19]. Low-*β* BiNbO_4_ powders were prepared by citrate and Pechini methods. Bi(NO_3_)_3_·5H_2_O was first dissolved in Nb-CA. Then the solution was kept stirring at 60 °C, and ammonia was added to adjust the pH value to 7–8. Finally, a stable solution was obtained. The above is the citrate method process; for the Pechini method, another step is added: after the stable and transparent solution was obtained, EG was dropped as chelating agent and stirring at 80 °C to promote the polymerization between metal CA and EG. The two kinds of solutions were finally dried at 180 °C to evaporate the solvent and calcined at various temperatures from 500 to 900 °C for 3 h with a ramp rate of 3 °C min^−1^.

The structure of the thermally treated powders was characterized by X-ray diffraction (XRD) with Cu *Kα* radiation. The grain sizes of the powders were examined using transmission electron microscopy (TEM). The photocatalytic activity of the BiNbO_4_ powders for the decomposition of MV was evaluated under irradiation of a 150-W Xe lamp at the natural pH value; the details have been described in the previous work, and the degradation process was monitored by an ultraviolet-visible near infrared (UV-vis-NIR) spectrophotometer [12].

## Results and Discussion

**Fig. 1:**
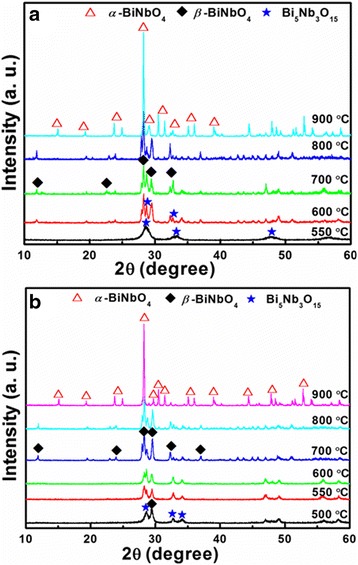
XRD patterns of BiNbO_4_ powders prepared by **a** citrate and **b** Pechini methods, respectively. The powders are thermally treated at different temperatures for 3 h

Figure 1 gives the XRD patterns of BiNbO_4_ powders prepared by citrate and Pechini methods. In Fig. 1a, it can easily be seen that there is no apparent *β*-BiNbO_4_ peaks at 550 °C, only an intermediate phase of Bi_5_Nb_3_O_15_ is formed. With the temperature increase, Bi_5_Nb_3_O_15_ transformed to *β*-BiNbO_4_ gradually and pure Low-*β* BiNbO_4_ is obtained at 700 °C. Due to the polymerization effect between CA and EG in the Pechini process, the appearance of Low-*β* BiNbO_4_ seems much easier, as shown in Fig. 1b. Even at 500 °C, Low-*β* BiNbO_4_ has been formed and coexisted with the Bi_5_Nb_3_O_15_ phases. At 550 °C, quite different from that of the citrate method, Low-*β* BiNbO_4_ is the major phase and pure Low-*β* BiNbO_4_ is also obtained at 700 °C. Low-*β* BiNbO_4_ is thermodynamically unstable and transforms to *α* phase at 900 °C, the same as in other literatures [15]. The differences of the phase formation process can be attributed to the polymerization effect between CA and EG. In the citrate process, the solution consists of Nb-CA and Bi-citrate (Bi-CA) metal complexes and is weakly interconnected by van der Waals or hydrogen bonding; while for the Pechini process, Nb-CA and Bi-CA were coordinated by the polymerization effect between CA and EG to form a homogeneous multicomponent gel, which is crucial for complex oxide preparation [18].

**Fig. 2:**
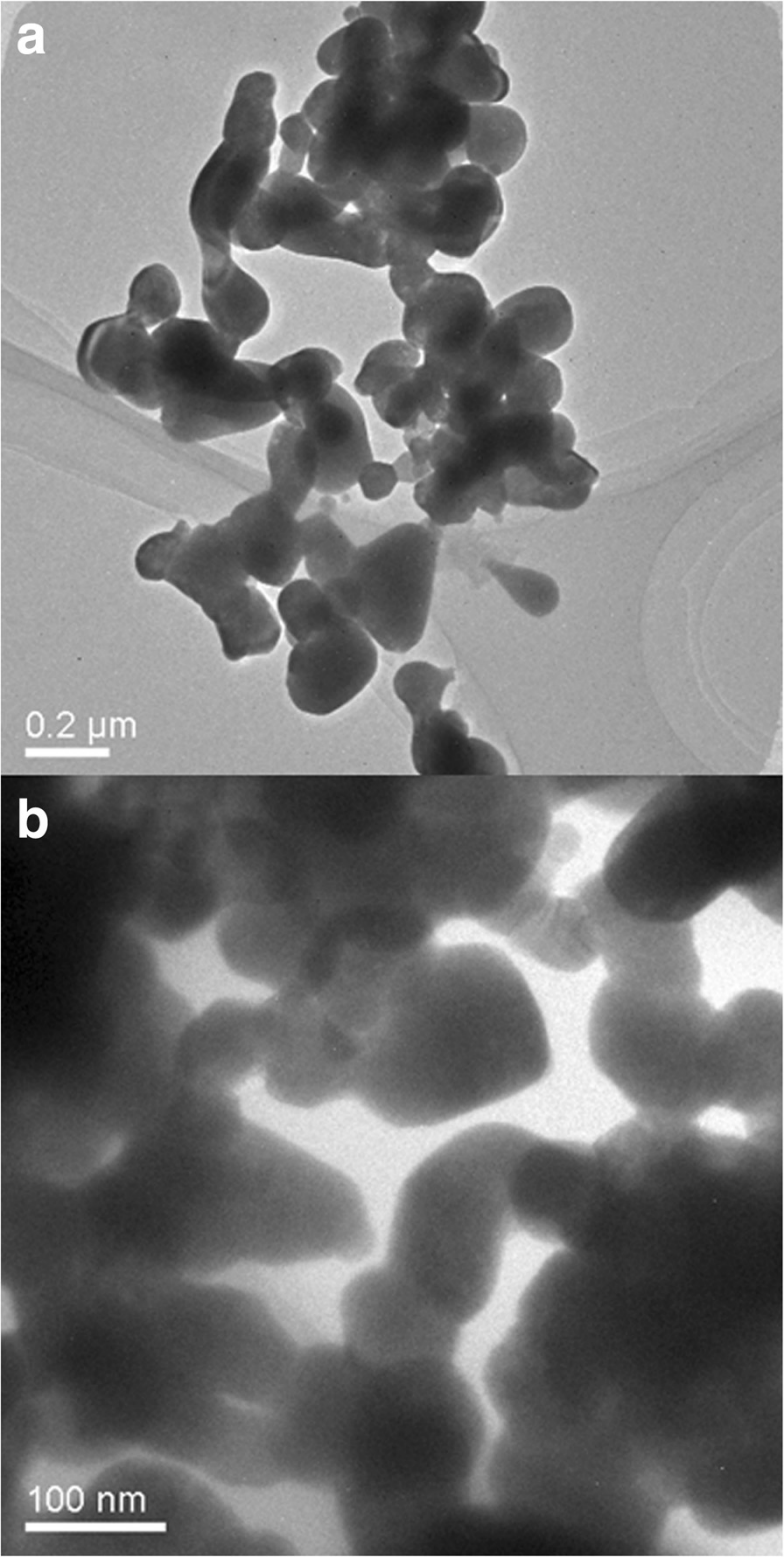
TEM images of Low-*β* BiNbO_4_ powders prepared by **a** citrate and **b** Pechini methods

The polymerization is beneficial to obtain smaller particles, as seen in the TEM imagines of Low-*β* BiNbO_4_ prepared by the two methods in Fig. 2. The particle size is between 70 and 100 nm for the Pechini method, smaller than that of the citrate method, about 150 nm, while the shape of Low-*β* BiNbO_4_ powders seems irregular for both methods due to the uncompleted crystal evolution.

**Fig. 3:**
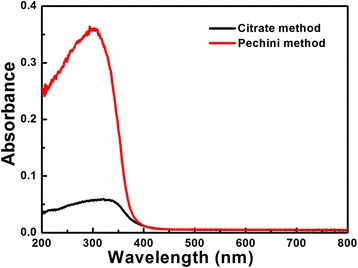
UV-visible absorption spectra of Low-*β* BiNbO_4_ powders prepared by citrate and Pechini methods

The room-temperature UV-vis absorption spectra of Low-*β* BiNbO_4_ powders are recorded in Fig. 3. A pressed BaSO_4_ powder was used as a reference, and the absorption spectra of the data were transformed from the diffuse reflection spectra according to the Kubelka-Munk (K-M) theory. Because Low-*β* BiNbO_4_ is the indirect band gap semiconductor, the relation between the absorption edge and photon energy (*hν*) can be written as follows:

1 $$ {\left(\alpha h\nu \right)}^{1/2}=A\left(h\nu -{E}_g\right) $$

where *A* is the absorption constant of the materials of indirect band gap semiconductor. The absorption coefficient (*α*) is determined from the scattering and reflectance spectra according the K-M theory. The energy band gaps (*E*_*g*_) of the catalysts prepared by the citrate and Pechini methods are estimated to be 2.85 and 3.1 eV, respectively, based on Eq. (1). It seems that the former catalyst can absorb the visible light better. It is consistent with the appearance of the Low-*β* BiNbO_4_ catalyst prepared by the citrate method, which is pale yellow, indicating that this material indeed absorbs the visible light to some extent, while the appearance of the sample prepared by the Pechini method is white. The larger band gap with Low-*β* BiNbO_4_ powders prepared by the Pechini method can be attributed to their smaller grain size and insufficient growth of grain.

**Fig. 4:**
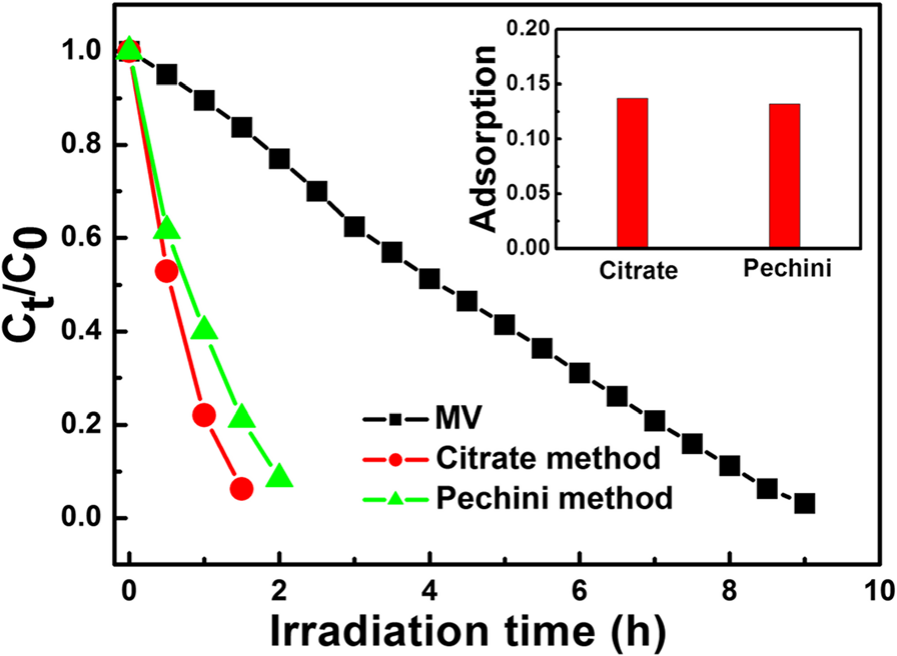
Photodegradation of MV with respect to the irradiation time using Low-*β* BiNbO_4_ powders exposed to visible light; the *inset* shows the adsorption ability of Low-*β* BiNbO_4_ powders after the suspension was agitated for 1 h in the absence of light to achieve the equilibrium adsorption

The visible-light photocatalytic activity of pure Low-*β* BiNbO_4_ powders has been investigated under visible-light irradiation (*λ* > 400 nm), as shown in Fig. 4. MV is adopted as a representative organic dye to evaluate the photocatalytic performance. *C*_0_ is the initial concentration of MV, and *C*_t_ is the equilibrium concentration of MV at visible-light irradiation time.

For BiNbO_4_ particles, the mechanism of MV degradation under visible-light irradiation involves photocatalytic and photosensitization pathways, and the latter has a dominant role in the degradation [12]. Both catalysts exhibit excellent performance of visible-light degradation of MV, compared with the degradation of MV without a catalyst. The Low-*β* BiNbO_4_ powder prepared by the citrate method shows a better degradation rate of about 1 h to decompose 80 % of MV. The better photocatalytic performance can be attributed to its smaller band gap and better crystallinity than that of catalysts prepared by the Pechini method. Better crystallinity plays an important role in the degradation of MV, corresponding to the adsorption ability test of BiNbO_4_ powders, as seen in the inset in Fig. 4. Though the catalysts prepared by the Pechini method are with a smaller particle size, the adsorption ability is equal to that of catalysts by citrate method.

In the heterogeneous photocatalytic studies, the photocatalytic degradation rate of the most organic compounds can be described by the modified Langmuir-Hinshelwood kinetics model [20, 21]. The photocatalytic degradation of MV using Low-*β* BiNbO_4_ powders obeys the pseudo-first-order kinetics. The representation of the rates of photodegradation of MV is given by

2 $$ \ln {C}_{\mathsf{t}}=-kt+ \ln {C}_{\mathsf{0}} $$

This equation can be modified to demonstrate linearity of data, if the Eq. (2) is given by

3 $$ \ln \left({C}_0/{C}_t\right)=kt $$

where *k* is the constant of the pseudo-first-order rate.

**Fig. 5:**
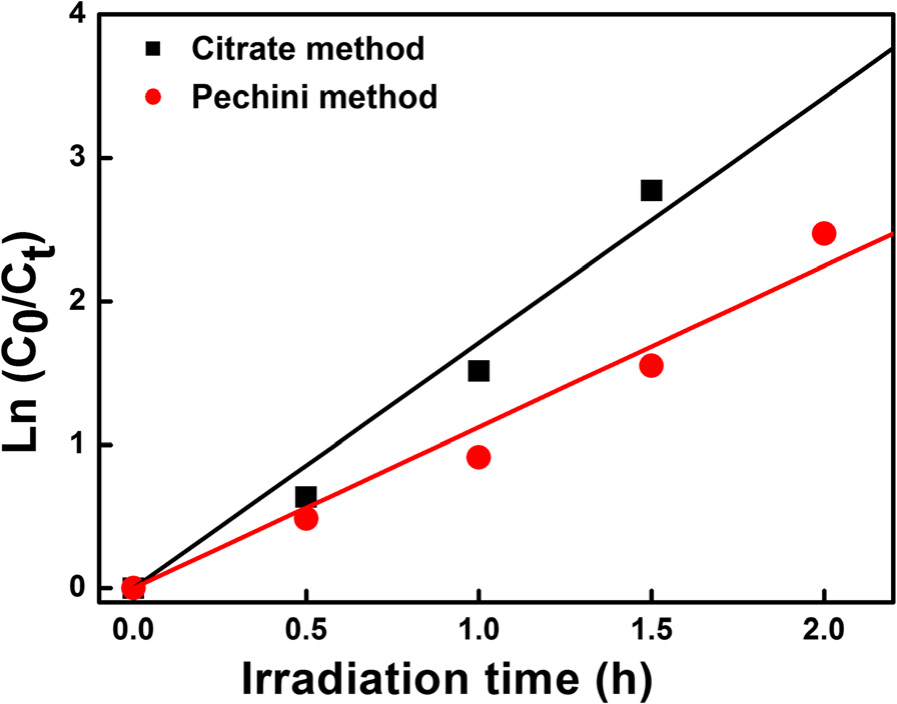
Kinetic fit for the photodegradation of MV in the presence of Low-*β* BiNbO_4_ powders prepared by citrate and Pechini methods

A plot of ln(*C*_0_/*C*_t_) versus the visible irradiation time for the MV photodegradation catalyzed by Low-*β* BiNbO_4_ powders is shown in Fig. 5. A linear relation between ln(*C*_0_/*C*_t_) and the irradiation time has been confirmed, which implies that the photodegradation of MV follows the first-order kinetics. The obtained first-order rate constant (*k*) and half-time (*t*_1/2_) are 2.85 × 10^−2^ min^−1^ and 24.3 min and 1.87 × 10^−2^ min^−1^ and 37 min, respectively. The higher the first-order rate constant is, the more outstanding the photocatalytic performance is. So the degradation of MV using Low-*β* BiNbO_4_ powders prepared by the citrate method shows higher *k* and smaller *t*_1/2_.

The effect of operating parameters such as the amount of catalyst loading, pH value, and the additive H_2_O_2_ concentration on the photocatalytic performance of Low-*β* BiNbO_4_ powders prepared by the citrate method has been investigated in our former work [12]. It shows that the optimal operation conditions are catalyst loading of 1 g L^−1^, pH value of 8, and the additive H_2_O_2_ concentration of 2 mmol L^−1^.

**Fig. 6:**
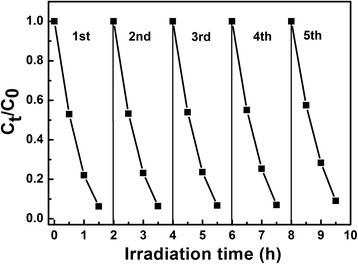
Cycling photodegradation of MV over Low-*β* BiNbO_4_ powder prepared by citrate method under visible light irradiation

To evaluate the reusability and stability of the as-synthesized photocatalysts, five cycles of photodegradation of MV are conducted taking Low-*β* BiNbO_4_ powder prepared by citrate method as typical samples. Figure 6 shows no obvious performance loss after five cycling runs of photodegradation of MV, which indicates that the Low-*β* BiNbO_4_ photocatalyst is an efficient visible light-driven photocatalyst with good reusability for potential practical applications in wastewater treatment.

## Conclusions

Low-*β* BiNbO_4_ powders were prepared by citrate and Pechini methods using homemade water-soluble niobium precursors. The addition of ethylene glycol and the resultant polymerization effect on the synthesis and visible-light photocatalytic performance of Low-*β* BiNbO_4_ powders were investigated. The polymerization effect is beneficial to lower the phase formation temperature and obtain smaller particle catalysts. Both methods can synthesize catalysts with excellent performance of visible-light degradation of MV. The Low-*β* BiNbO_4_ powder prepared by the citrate method shows a better degradation rate of about 1 h to decompose 80 % of MV and also displays good photocatalytic stability. The photodegradation of MV under the visible-light irradiation followed the pseudo-first-order kinetics according to the Langmuir-Hinshelwood model, and the obtained first-order rate constant and half-time are 2.85 × 10^−2^ min^−1^ and 24.3 min, respectively. The better photocatalytic performance of BiNbO_4_ powders prepared by the citrate method can be attributed to its smaller band gap and better crystallinity.
